# Butyrylcholinesterase-Loaded Liposomes and Polymersomes: Catalytic Parameters for Three Types of Substrates

**DOI:** 10.3390/ijms27010190

**Published:** 2025-12-24

**Authors:** Zukhra Shaihutdinova, Svetlana Batasheva, Patrick Masson, Tatiana Pashirova

**Affiliations:** 1 Institute of Fundamental Medicine and Biology, Kazan Federal University, 18 Kremlyovskaya St., 420008 Kazan, Russia; 2 Arbuzov Institute of Organic and Physical Chemistry, FRC Kazan Scientific Center, Russian Academy of Sciences, Arbuzov Str. 8, 420088 Kazan, Russia

**Keywords:** bioscavenger, butyrylcholinesterase, enzyme nanoreactor, liposome, organophosphorus compound, polymersome

## Abstract

The nano-technological approach and supramolecular chemistry principles relation to the encapsulation of enzymes pave the way for creating next-generation nano-system-functionalized nano-compartments. The most promising approach for prophylaxis and the treatment of organophosphate (OP) poisoning is the use of stable, bioavailable nano-compartments containing OP-scavenging enzymes. Such enzymes, like butyrylcholinesterase (BChE), wild type and mutants, could also be used for the detoxification of other poisonous esters. There are two types of IRD-labeled human BChE-containing nano-scavengers: PEGylated liposomes and polyethyleneglycol–polypropylenesulfide polymersomes, which were developed with diameter close to 100 nm. BChE-polymersomes have higher encapsulation efficiency (95%) and slower release rate of enzymes (more than 7 days) compared to BChE-liposomes. The catalytic properties of encapsulated enzymes were analyzed for nano-compartment formulations, lipophilicity, the structure of block copolymers, and for different ester substrate polarity: positively charged butyrylthiocholine iodide, neutral phenyl acetate, and negatively charged aspirin. The highest *k_cat_* (more than three times) compared to non-encapsulated BChE was for polymersomes based on diblock PEG-PPS polymersomes towards the neutral phenyl acetate substrate.

## 1. Introduction

Currently, biocatalytic approaches are moving beyond simple enzyme immobilization strategies toward nano-technologies. The nano-technological approach and supramolecular chemistry principles for the encapsulation of enzymes leads to next-generation nano-systems, namely those starting from simple colloidal carriers and targeted drug delivery systems to functionalized nano-compartments [1,2,3]. Catalytic reactions and chemical processes take place inside of such functionalized nanoparticles. Understanding the fundamental functioning principles of such nanomaterials will make it possible to regulate the properties of artificial organelles, cells, and organs [4,5,6,7]. Moreover, this approach leads to new generation of high-tech nano-systems [8,9,10,11,12].

Our group’s research focuses on biocatalytic nano-systems, specifically the encapsulation and functionality of bioscavengers against organophosphorus compounds (OP) (butyrylcholinesterase (BChE) and phosphotriesterase) as medical countermeasures against OP poisoning [13,14,15,16], which are aimed at creating the next generation of novel nano-antidotes toward OPs. This new approach was shown to be promising both in the treatment and prophylaxis of organophosphate poisoning. This approach implements encapsulated either stoichiometric or pseudo-catalytic and catalytic bioscavengers [17,18]. At this point, it is important to mention that BChE is an important esterase involved in the detoxification of numerous carboxyl esters as drugs, natural poisons, and narcotics [14,19]. Previous works showed that butyrylthiocholine (BTC), the model carboxyl ester substrate of BChE, can be degraded in nanoreactors [15,20]. Therefore, BChE nanoreactors could also be used for the treatment of acute poisoning by carboxyl-ester substrates.

Nano-bioantidotes or nano-bioscavengers are stable, bioavailable nano-compartments containing one or several enzymes embedded in nano-vesicles sealed by a polymeric membrane. The limited reaction volume for encapsulated enzymes results in a high probability of interaction between the enzyme and the substrate, and access to the enzyme catalytic center. However, at the moment, the kinetic principles of enzyme reactions inside nano-compartments are still not fully understood. It is known that the limited volume inside nano-compartments provides a high concentration of encapsulated enzyme molecules and explains the high reactivity [21]. In other words, a high concentration of embedded highly reactive enzyme is one of the key factors causing fast reactions taking place inside nano-compartments [22].

The fast penetration of a substrate through the membrane of nano-compartment is also very important for high catalytic efficiency. However, the chemical structure of substrates (charge, hydrophobicity, and hydrophilicity) may affect diffusion inside nanoreactors. In addition, the catalytic behavior of the encapsulated enzyme may differ from the behavior of the enzyme in solution. For example, the increase in enzyme *k_cat_* inside the compartment compared to the enzyme in free solution was explained by the increased probability of the collisions of enzyme with the substrate and with the nano-compartment membrane [23,24]. However, a change in the enzyme’s molecular dynamics cannot be ruled out. Two physico-chemical properties have also to be considered: (i) the lipophilicity of the membrane and (ii) the structure of substrate, namely its hydrophilic–lipophilic balance and the electric charge. It is known from the literature that free diffusion into liposomes is observed only for small and uncharged substrates [25,26] without changing the catalytic parameters of enzymatic reactions [24]. Difficulty in the diffusion of substrates to the enzyme due to poor permeability across the nanoparticle membrane [27] can be caused by highly hydrophobic membranes [28], or the presence of phosphatidylglycerol in lipid membrane [29]. To shed light on these processes, two types of nano-containers were considered, and the hydrolysis kinetics of different structurally related carboxyl ester substrates of BChE were investigated. The enzyme was IRD-labeled, with a view to further in vivo studies.

In the present study, two types of IRD-labeled BChE-containing nanoscavengers, liposomes and polymersomes, were considered. The lipophilicity of the polymersome membrane was varied by changing the structure of amphiphilic di- and triblock copolymers polyethyleneglycol–polysulfide (molecule weight and length of the hydrophobic block). The catalytic parameters of the BChE-catalyzed reaction were evaluated and compared for different nano-compartments by varying the membrane’s lipophilicity for the penetration of model carboxyl ester substrates of different charges: a positively charged ester (butyrylthiocholine iodide, BTC), a negatively charged ester (aspirin), and a neutral ester (phenyl acetate, PhA).

## 2. Results

### 2.1. Preparation and Characterization of BChE-IRD-Containing Nano-Scavengers

**Figure 1:**
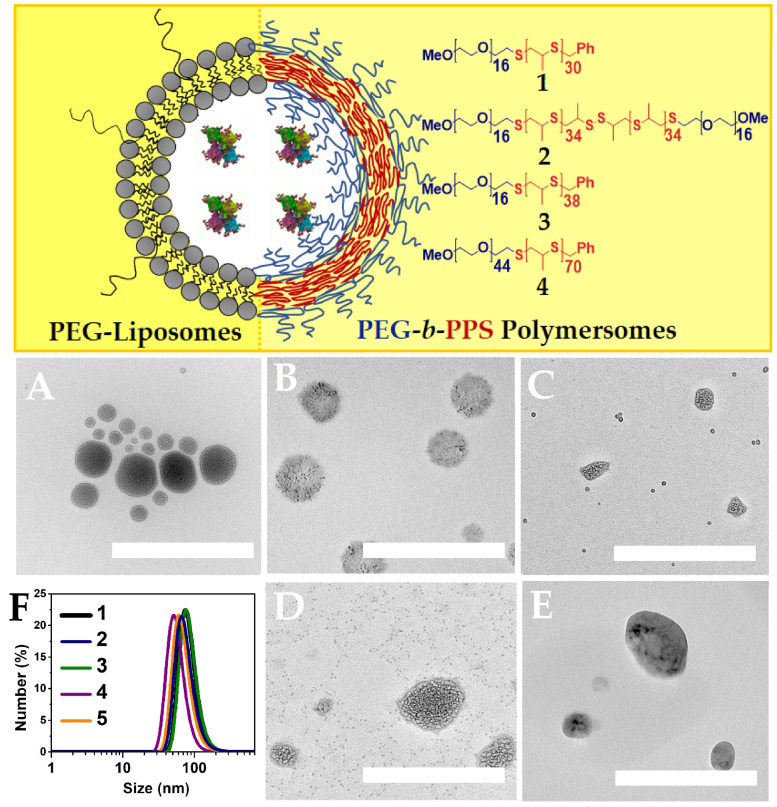
Two types of BChE-containing compartments (nano-scavengers): PEGylated liposomes and PEG-PPS polymersomes, where 1—di-PEG-*b*-PPSwith *f_PEG_* = 0.24 and Mn mPEG = 750, 2—triblock-PEG-*b*-PPS with *f_PEG_* = 0.23 and Mn mPEG = 750, 3—diblock-PEG-*b*-PPS with *f_PEG_* = 0.21 and Mn mPEG = 750, and 4—diblock PEG-*b*-PPS with *f_PEG_* = 0.27 and Mn mPEG = 2000. TEM of BChE-containing PEG-liposomes after 2-month storage at 4 °C (**A**) and the PEG-*b*-PPS-polymersomes, PEG-*b*-PPS-1 (**B**), PEG-*b*-PPS-2 (**C**), PEG-*b*-PPS-3 (**D**), and PEG-*b*-PPS-4 (**E**). DLS (**F**) of BChE-containing liposomes (1), polymersomes-1 (2), polymersomes-2 (3), polymersomes-3 (4), and polymersomes-4 (5). Scale bar is 500 nm.

Human BChE is a temperature-sensitive enzyme. Therefore, to preserve the enzyme activity, a gentle encapsulation method was used. Film evaporation and hydration at temperatures not exceeding 45 °C were used. Two types of enzyme-containing compartments were chosen: PEGylated liposomes with a phospholipid membrane, and polymersomes based on amphiphilic polyethyleneglycol–polypropylenesulfide block copolymers (PEG-*b*-PPS) (Figure 1).

The selection and synthesis of PEG-*b*-PPS was based on their hydrophilicity parameter (*f_PEG_*). PEG-*b*-PPS with *f_PEG_* values range from 0.2 to 0.3, organizing nanoparticles with a “water bag” morphology or polymer vesicles. The polymer membrane varied depending on the following: (a) the structure of di-(PEG-*b*-PPS-1) or triblock copolymers (PEG-*b*-PPS-2), (b) the length of hydrophobic block PPS (PEG-*b*-PPS-3), and (c) the layer thickness (PEG-*b*-PPS-4).

The physico-chemical characteristics of IRD-BChE-containing polymersomes and liposomes are shown in Figure 1, Table 1, and Figures S1–S6 (Supplementary Materials).

DLS data show that the size is close to 100 nm and polydispersity does not exceed 0.22. The zeta potential is electronegative, because of the PEG molecules located on the outer surface of the nanoparticles. Then, the comparison of freshly prepared samples and samples after 2-month storage was performed. As we can see, polymersomes are more stable than liposomes upon storage. To confirm the preservation of the morphology and shape of both types of nano-compartments, liposomes and polymersomes, transmission electron microscopy (TEM) was used. TEM shows that all nano-compartments have a water–core structure. However, there is difference between PEG-liposomes and polymersomes. Thus, the membrane is observed for PEG-liposomes (Figure 1A and Figure S1). A “hairy coat”, most likely caused by PEG, is observed for polymersomes (Figure 1B and Figure S1). Also, polydispersity is observed for PEG-liposomes, and polymersomes-2 (Figure 1C and Figure S1) and polymersomes-3 (Figure 1D and Figure S1). It is assumed that liposome polydispersity is due to the fusion of liposomes’ nanoparticles as a function of time. Also, PEG-*b*-PPS polymersomes-2 and 3 may form mixed associates of about a 10 nm size with BChE through non-covalent interactions. This leads to slight decrease in size over time (DLS data), due to the formation of mixed enzyme–polymer associates. In addition, we also observed the formation of small associates with BChE for PAA-PS block copolymers [20].

The encapsulation efficiency was found by spectrophotometry at λ = 778 nm for IRD-BChE (Figure S7, Supplementary Materials). The EE (%) for polymersomes (ranging from 88 to 95%) is higher than for liposomes (77 and 85%). Polymersomes-2 and 3 with a three-block structure and a thicker membrane a have higher EE. Then, it can be stated that the lowest EE of liposomes is associated with lower LC (1%) compared to polymersomes (3%).

**Figure 2:**
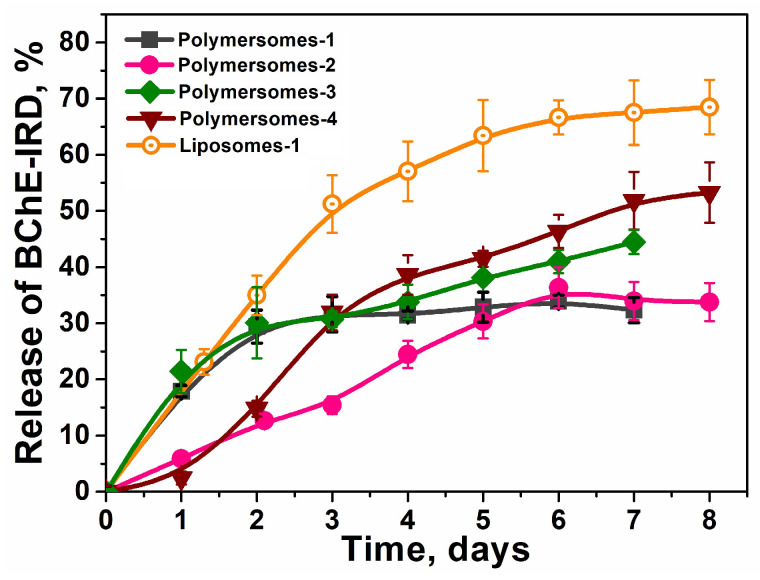
Release of BChE from nano-systems as a function of time in 10 mM Tris/HCl buffer, pH 7.4, 37 °C. C_BChE_ = 0.16 mg/mL. Data are mean values ± SE of triplicate measurements.

Nano-scavengers must circulate in the bloodstream, keeping loaded enzymes for a long time. Thus, it was important to find and compare the enzyme release time of all systems. The release of enzymes from all prepared systems was monitored by spectrophotometry at λ = 778 nm for IRD-BChE (Figure 2 and Figure S8). The most promising nano-scavengers are polymerosome-2. A slower release rate was established, reaching about 30% after 6 days. Polymersomes-1 released 30% o enzymes in the first two days, and after this period of time, no release was observed. Liposomes are the fastest-releasing enzyme nano-systems, with 30% of BChE released in less than two days, and then about 70% of released BChE was observed on the sixth day.

For release experiments, samples were placed in a large volume of non-sterile buffer. After two months of release at 25 °C, no enzyme activity was detected in this buffer. However, when stored at +4 °C, nano-system samples remain stable and retain 100% activity at least for two months (Figure S9). However, after a year and half at +4 °C, enzyme activity dropped to 50%. When enzyme nano-systems were stored at room temperature for 24 h, their activity remained unchanged, but after three days, their activity dropped to 90% of initial value (Figure S9). Encapsulation protects the active enzyme against degradation.

### 2.2. Kinetic Studies of BChE-Loaded Nano-Systems

**Figure 3:**
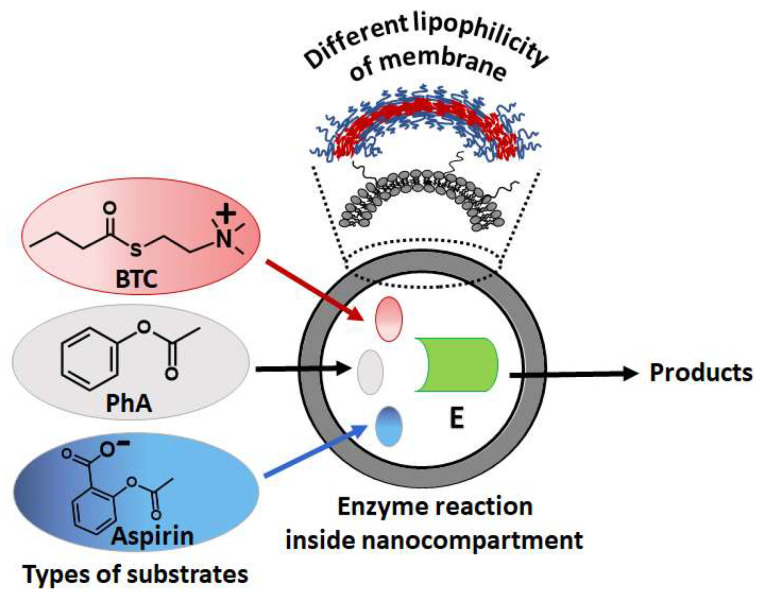
BChE catalyzed hydrolysis of three types substrates: positively (BTC) and negatively (aspirin) charged, and neutral (PhA) esters.

The study of the kinetics of the BChE-catalyzed hydrolysis of substrates in nano-systems was carried out by varying the type, structure, and thickness of nano-system membranes. Three types of ester substrates were chosen: positively charged (BTC), negatively charged (aspirin), and neutral (PhA) (Figure 3).

**Figure 4:**
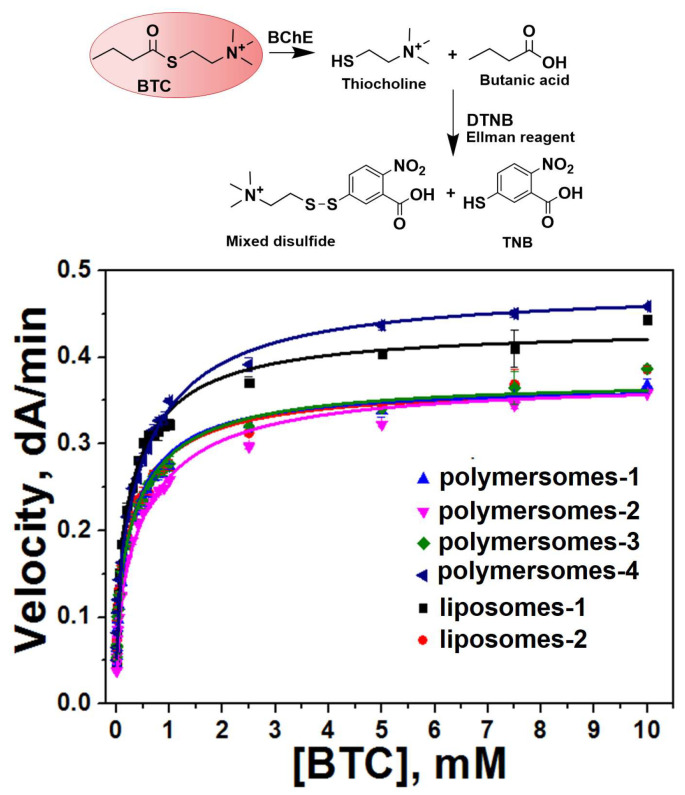
Rate of BTC hydrolysis by BChE-containing nano-systems versus BTC concentration in 10 mM Tris/HCl buffer, pH 7.4, 25 °C. C_BChE_ = 0.2 nM, C_active sites of BChE_ = 0.8 nM. Data are mean values ± SE of triplicate measurements. r^2^ = 0.99 for all dependencies.

The concentration of enzymes inside the nanoparticles was determined by purifying nanoparticles from free non-encapsulated enzymes, and taking into account the encapsulation efficiency. The enzyme concentration inside nano-compartments is high. According to our previous work [13], the enzyme concentration is approximately 1 mM (experimental) or 40 molecules/nanoparticle (calculated value) for all types of nano-compartments, regardless the diameter of these compartments (Table 1). This significantly exceeds the concentration of substrate penetrating across the membrane. This condition creates a reverse concentration gradient for the substrate, triggering the rapid enzyme-mediated hydrolysis of substrate molecules. The hydrolysis of BTC was recorded by the Ellman method at 412 nm (Figure S10). It is known that butyrylcholinesterase behavior with positively charged substrates such as BTC is not Michaelian, and shows activation by excess substrate beyond 1 mM [30]. As seen, the encapsulation of BChE in different types of nano-compartments does not change the reaction mechanism with the water-soluble substrate BTC (Figure 4). All nano-systems showed ascending hyperbolic dependencies and were fitted to the Radic equation model [30]. The catalytic parameters of the free enzyme and the enzyme loaded into different types of nano-compartments are shown in Table 2.

We see that all parameters changed after the encapsulation of the enzymes. The catalytic efficiency (*k_cat_*/*K_m_*) was found to depend on the structure of block copolymers. It is decreased most significantly (by three times) in the case of polymersomes-2 prepared by the triblock copolymer. *k_cat_*/*K_m_* is comparable for polymersomes from diblock copolymers and liposomes. It should be noted that for all systems, *k_cat_* decreases, except in liposomes-1. At the same time, *K_m_* increases in all nano-systems. The maximum increase is observed for liposomes-1.

The next step was to study the influences of polarity and the charge of substrate on its penetration into the nano-compartment and the catalytic activity of the enzyme toward each substrate. Polymersomes-1 were chosen as nano-compartments. For this purpose, water-soluble substrates with practically identical lipophilicity but displaying different polarity were selected: phenyl acetate (PhA) and aspirin. Thus, their calculated LogP values (by Molinspiration program) rank according to the following sequence:

BTC (−1.79) < aspirin (1.43) ≤ PhA (1.49).

Their topological polar surface areas (TPSA) calculated by Molinspiration program are as follows:

BTC (17.07) < PhA (26.30) < aspirin (63.6)

**Figure 5:**
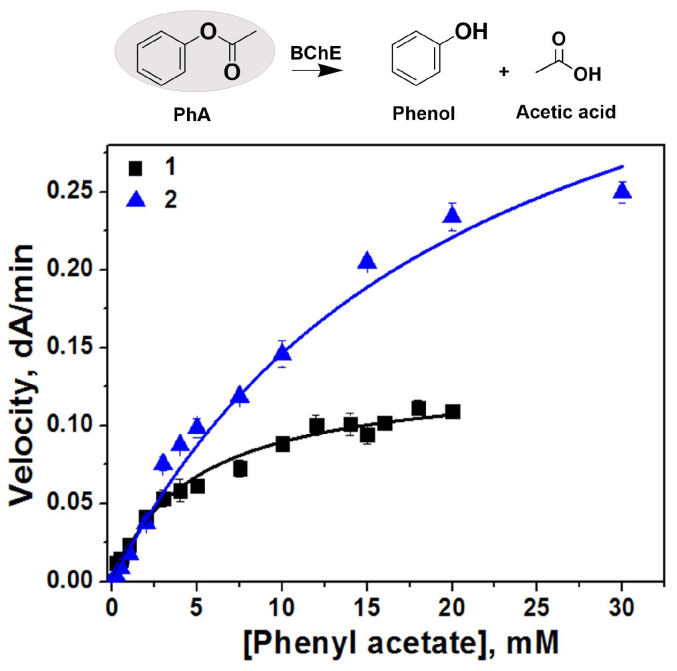
Rate of phenyl acetate hydrolysis by BChE (1) and BChE-containing polymersomes-1 (2) versus phenyl acetate concentration in 10 mM Tris/HCl buffer, pH 7.4, 5% of methanol, C_BChE tetramer_ = 1 nM, C_active sites of BChE_ = 4 nM, 25 °C. Data are mean values ± SE of triplicate measurements. r^2^ = 0.99 (curve 1); 0.98 (curve 2).

Unlike positively charged substrates like BTC, the catalytic behavior of BChE with phenyl acetate (Figure S11, Supplementary Materials) and aspirin (Figure S12, Supplementary Materials) is Michaelian. BChE-loaded polymersomes-1 showed the classical Michaelian hyperbolic dependency with PhA (Figure 5) and plots were fitted to the Michaelis–Menten equation (Equation (1)).

(1) $$v=\frac{k_{cat}\left[E\right]\left[S\right]}{1+K_{m}/\left[S\right]}$$

From Table 2 it can be seen that unlike positively charged BTC, in the case of the uncharged substrate PhA, the catalytic effect of nano-systems is mild, and catalytic parameters are close to parameters of the free enzyme. BChE-containing polymersomes-1 shows a three-times higher *k_cat_* for PhA than free BChE, and the binding affinity (1/*K_m_*) is reduced (by four times) compared to the free enzyme. However, the specificity constant (*k_cat_*/*K_m_*) was not significantly changed.

**Figure 6:**
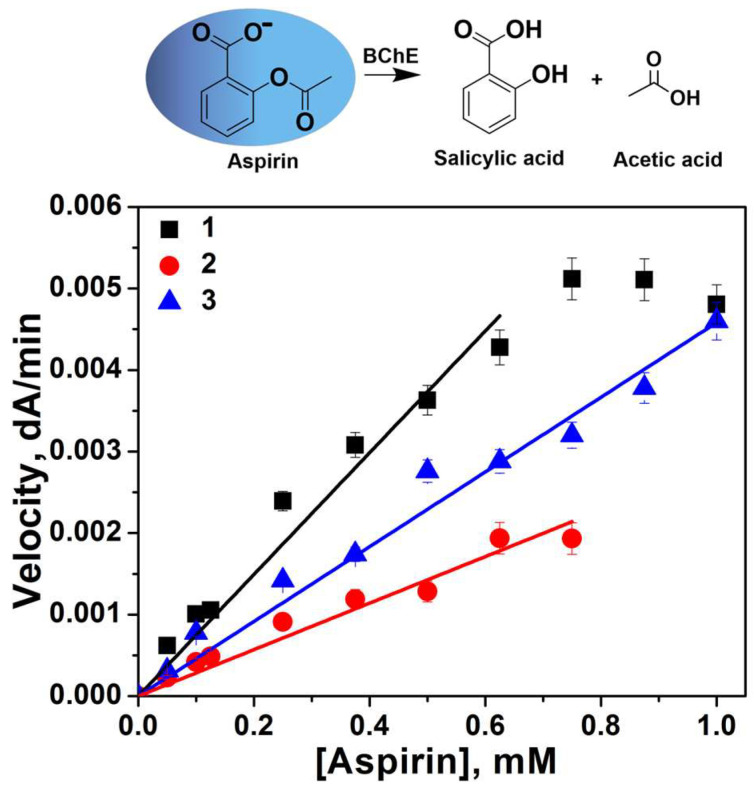
Rate of substrate Aspirin hydrolysis by free BChE (1) and BChE-loaded polymesomes-1 (2 and 3) versus aspirin concentration in 10 mM Tris/HCl buffer, pH 7.4, with 10 mM CaCl_2_ (1 and 2) and in 100 mM Tris/HCl buffer, pH 7.4, with 50 mM CaCl_2_ (3), 25 °C. C_BChE_ = 1.6 nM, C_active sites of BChE_ = 6.25 nM, 5% of methanol. Data are mean values ± SE of triplicate measurements. r^2^ = 0.98 (lines 1 and 3); 0.99 (line 2).

The aspirin hydrolysis was monitored only at the beginning of the Michaelis–Menten plot due to limitations of aspirin solubility in buffer solution (Figure 6).

**Table 2:** Catalytic parameters of substrate hydrolysis by free and BChE-encapsulated nano-systems; 10 mM Tris/HCl buffer, pH 7.4. Data are mean values ± SE of triplicate measurements.

Scavengers	Type of Substrate	*k_cat_*, min^−1^	*K*_*m*_, μM	*k_cat_*/*K*_*m*_ × 10^6^, M^−1^min^−1^	*K*_*ss*_, μM	*b*
Free BChE	BTC	16,800 ± 2100	4.9 ± 1.8	3410 ± 1690	503 ± 79	3.8 ± 0.4
BChE-polymersomes-1		10,400 ± 500	7.2 ± 0.9	1440 ± 30	410 ± 25	3.5 ± 0.2
BChE-polymersomes-2		10,100 ± 500	9.2 ± 1.4	1090 ± 220	670 ± 40	3.7 ± 0.2
BChE-polymersomes-3		13,900 ± 520	8.4 ± 0.9	1660 ± 240	670 ± 30	3.4 ± 0.1
BChE-polymersomes-4		12,400 ± 680	7.3 ± 0.9	1690 ± 320	530 ± 50	3.0 ± 0.2
BChE-liposomes-1		16,600 ± 1100	12.3 ± 1.7	1350 ± 270	524 ± 68	2.6 ± 0.2
BChE-liposomes-2		13,400 ± 1000	8.7 ± 1.4	1540 ± 350	520 ± 76	2.7 ± 0.2
Free BChE *	PhA	22,630 ± 890	5.3 ± 0.6	4.28 ± 0.66	-	-
BChE-polymersomes-1 *		74,760 ± 9840	20.8 ± 3.8	3.58 ± 1.12	-	-
Free BChE **	Aspirin ****	-	-	0.341 ± 0.015	-	-
BChE-polymersomes-1 **		-	-	0.130 ± 0.006	-	-
BChE-polymersomes-1 ***		-	-	0.209 ± 0.006	-	-

* 10 mM Tris/HCl buffer, pH 7.4, containing methanol 5% (vol.); ** 10 mM Tris/HCl buffer, pH 7.4, with 10 mM CaCl_2_; *** 100 mM Tris/HCl buffer, pH 7.4, 50 mM CaCl_2_; and **** because of the limited solubility of aspirin in buffers, steady-state kinetics with this substrate were performed at low aspirin concentration, less than *K_m_*. Therefore, only the *k_cat_*/*K_m_* values were determined from the slopes of initial linear portions of Michaelis–Menten curves, *v* = (*k_cat_*/*K*_m_)[*E*][*S*] (Figure 6).

Under these conditions, the investigated maximum substrate concentration was much less than *K_m_*, and the rate equation (Equation (1)) reduces to *v* = (*k_cat_*/*K_m_*)[*E*][*S*]. Thus, slope of linear plots directly provides the value of *k_cat_*/*K_m_*. As for a positively charged substrate BTC, in the case of aspirin, there is a decrease in the catalytic specificity (*k_cat_*/*K_m_*) for BChE-loaded polymersomes-1 compared to free enzymes.

## 3. Discussion and Conclusions

It is becoming clear that the nano-scavenger approach is one of the most promising methods for the in vivo detoxification of harmful compounds, in particular OPs [31,32,33,34,35]. Only nano-scavengers are capable of working in a comprehensive manner, by (i) overcoming biological barriers, (ii) circulating in the bloodstream for a long time, and (iii) most importantly, fast binding and reacting with organophosphorus compounds for quasi-immediate neutralization into nontoxic products. Such an effective process in the bloodstream prevents the transfer of toxic molecules to physiological targets, e.g., nervous system acetylcholinesterase in the case of acute poisoning by OPs. The gold standard for long-term stability in the blood circulation is either PEGylation, as we did, or the production of stealth nanoparticles [36,37]. Despite its shortcomings, alternatives, and the presence of other different stealth polymers [38], PEG is approved by the US Food and Drug Administration and other national sanitary agencies, and is still the preferred polymer for the surface modification of biomedical products. For possible future applications in nanomedicine, PEG-liposomes and PEG-b-PPS polymersomes were, thus, selected as nanocomponents for the loading of human BChE. Still only few protein-loaded liposome systems are commercially available. When considering polymersomes as alternative to liposomes, the clinical advantages of polymersomes over liposomes need to be established [39]. A clear stability superiority of polymersomes over liposomes was proposed as a criterium. However, only a few works have compared these two types of nanoparticles [40,41,42]. Therefore, comparison of freshly prepared and 2-month-old samples was performed, using the DLS method and TEM. It was found that polymersomes are more stable than liposomes.

The volume and shape of the nano-compartment plays a major role in activity, which determines the concentration of the included enzyme molecules [21,22,43]. All prepared BChE-loaded nano-systems have close volume and shape, except polymersomes-2. Also, the formation of self-assemblies with a size smaller than 30 nm may affect the activity of BChE [20]. We observed that for triblock-PEG-*b*-PPS-2 and diblock PEG-*b*-PPS-3, increasing *K_m_* and *K_ss_* most likely results from the formation of small self-assemblies of size 10 nm. Such a small nanobody may impose structural and dynamics constraints on the encapsulated enzyme, affecting its properties.

If the diffusion of the substrate/products does not change despite potential barriers due to the crossing of the nanoreactor envelope, *K_m_* should not depend on the encapsulation of the enzyme since this parameter is an intrinsic property of the enzyme [44]. However, several studies have shown that *K_m_* decreases for encapsulated enzymes compared to free enzymes, indicating an increase in affinity for substrates [45,46]. This beneficial effect results from the fact that the probability of interaction between the enzyme and the substrate is increased in a limited space. In particular, it was reported that a limited crowding environment can lead to structural/conformational changes in enzymes, resulting in decreased enzyme activity and increased substrate affinity [47].

In our work, the *K_m_* of BChE for substrates in nano-systems is higher than that for the free enzyme in all cases. Although no direct permeability measurements of substrates across nanoreactor membranes were performed, it can be tentatively stated that *K*_m_ increases may result from limited diffusion through the membrane of nano-compartments. This explanation was proposed in the work [48]. Taking into account a finding from our early works that the hydrolysis product (p-nitrophenolate) formed inside polymersomes is released via a burst mechanism [13,14], it may be concluded that the polymersome membrane has a good permeability compared to the liposomal membrane for small molecules such as p-nitrophenolate. BTC diffuses worse through the liposomal membrane than through the polymeric membrane. It is likely that the liposomal membrane, containing cholesterol, is more rigid than the polymer membrane. Based on earlier data obtained by the authors of [49], cholesterol does not change the kinetic parameters of the enzyme reaction, but sharply alters the values of substrate permeability [49].

Therefore, in our case, electrostatic interactions between the positively charged substrate BTC and the phospholipid cannot be ruled out, which also hinders substrate’s diffusion into the nanoreactor core. This statement is in agreement with the reported literature data [25]. In addition, the binding of phospholipids to the enzyme surface can increase cholinesterase activity, as reported is in several studies [50,51]. Polymersomes-1 exhibited the lowest effect on *K_m_* and were chosen in order to study their influence on the catalytic properties of BChE with substrates differing in polarity and charge. Aspirin and phenylacetate have similar lipophilicity but different charges. It is clear that the catalytic specificity (*k_cat_*/*K_m_*) of encapsulated BChE is slightly higher for the neutral substrate than for aspirin compared to the catalytic specificity of the free enzyme for these substrates (Table 2). The differences in *k*_cat_/*K*_m_ are not statistically different for PhA, suggesting that confinement does not impair the catalytic efficiency of the enzyme for the neutral ester. For aspirin, the modest difference between the free and encapsulated enzymes could be attributed to the negative charge (interestingly, an opposite trend was observed for the positively charged substrate BTC; Table 2). However, without further studies, it would be premature to provide a molecular interpretation of the charge effect.

The mechanism of the release of hydrolysis products was not investigated. This is an important issue that deserves further investigations. Indeed, hydrolysis products can either accumulate inside nanoreactors or freely diffuse outside nanobodies. The fact that our kinetic studies did not show enzyme inhibition by products at high substrate concentrations suggests that products did not accumulate inside nanoreactors. However, at this point, it would be premature to generalize to all types of substrates/products.

In conclusion, in view of the use of BChE-based enzyme nanoreactors in medical countermeasures against OP poisoning, we have always to keep in mind that the encapsulated enzyme catalytic efficiency against OP molecules may depend on the hydrophobicity and neutrality/charge of the toxicant. Such a conclusion suggests that the association of several types of enzyme nanoreactors—differing in membrane polymeric structure—must be formulated to cover the whole spectrum of potential toxicants.

## 4. Materials and Methods

### 4.1. Chemicals

Lipoid S PC phosphatidylcholine from soybean (98.0%) was a gift from Lipoid GmbH (Ludwigshafen, Germany), Lipoid SPC-3 hydrogenated phosphatidylcholine (98.0%) from soybean was a gift from Lipoid GmbH (Ludwigshafen, Germany), and cholesterol (Ch, ≥95%, Acros Organics, Scheepsbouwersweg, The Netherlands), 1,2–distearoyl–sn–glycerol–3–phosphor ethanolamine-N-[methoxy(polyethylene glycol)-2000] (ammonium salt) (18:0 PEG2000PE, ≥98.0%, Avanti polar lipids Inc., Alabaster, AL, USA), Butyrylthiocholine iodide (BTC) and dithio-bis-nitrobenzoic acid (DTNB) were from Sigma-Aldrich, Saint-Louis, MO, USA. The stock solution of BTC (0.1 M) prepared in water was stored at −20 °C. The stock solution of DTNB (0.01 M) supplied with 1.5 mg/mL NaHCO_3_ was prepared in 10 mM Tris buffer, pH 7.4. The stock solution of phenyl acetate (PhA, 99%, Sigma-Aldrich, Saint-Louis, MO, USA) and acetylsalicylic acid (aspirin, 99%, Sigma-Aldrich, Saint-Louis, MO, USA) were prepared in methanol.

The details of PEG-*b*-PPS block copolymer synthesis are in our previous work [13,14,15]. Structure confirmation by ^1^H and ^13^C NMR spectroscopy and IR is in our previous works. The hydrophilicity parameters (*f_PEG_*) were calculated as Mw(PEG)/Mw(PEG) + Mw(PPS) by comparing the integral intensity of PPS methyl group protons to that of methoxy group protons of mPEG from ^1^H NMR spectrum.

All other chemicals and solvents were of chemical or bio-chemical grade. Ultra-purified water (18.2 MΩ cm resistivity at 25 °C) was produced from Direct-Q 5 UV equipment (Millipore S.A.S. 67120 Molsheim, France).

### 4.2. Enzyme

Highly purified human BChE was labeled with IRDye800CW NHS Ester (NIR Dye Pack, Doc #988-18083, LI-COR Biosciences, Lincoln, NE, USA). The purpose of enzyme IRD-labeling was to allow the easy and sensitive monitoring of the fate of the injected enzyme in animal models. In addition, the IRD-labeling allowed us to monitor the enzyme concentration at all encapsulation steps, using absorption spectrophotometry at 778 nm. The labeling does not alter the catalytic properties of the enzyme. The BChE activity of the pure preparation was 3178 units/mL which corresponds to 6.4 mg/mL. All the details regarding enzyme purification and NIR probe labeling are in our previous work [15]. The concentration of NIR-labeled enzyme (IRD800-BChE) active sites used for experiments was determined by titration with echothiophate; it was equal to 1 μM in 45 Units/mL of enzyme solution.

### 4.3. Preparation of BChE-Containing Nano-Systems

#### 4.3.1. BChE-Containing Polymersomes

Polymersomes based on amphiphilic PEG–*b*-PPS block copolymers were prepared by the film hydration method. PEG-PPS block copolymers were dissolved in 1 mL of ethanol:chloroform solution (1:1 ratio). The resulting homogeneous solution was kept in a water bath in the temperature range of 34 °C, until the complete evaporation of the alcohol and the formation of a thin film. Then, the BChE-IRD enzyme solution (10 mM Tris buffer, pH 7.4) was preheated to 37 °C and added to the film to hydrate the copolymer at a temperature of 37 °C. The PEG-*b*-PPS solution (0.5% *w*/*w*) containing BChE (C = 0.47 μM) was mixed using a magnetic stirrer (Ika, Staufen, Germany) at 750 rpm for 3 h at the temperature 37 °C and then for 6 h at the temperature 25 °C.

#### 4.3.2. BChE-Containing Liposomes

SPC (or SPC-3), Ch, and 18:0 PEG2000PE, at a molar ratio 10:5:1, were dissolved in 1 mL of ethanol (final total lipid 1.2% wt/wt). The homogeneous solution was kept in a water bath at 45 °C until complete organic solvent evaporation (overnight) to obtain a thin lipid film. Then IRD-BChE solution (10 mM Tris buffer, pH 7.4) was pre-heated to 45 °C and added to the film to hydrate the lipid film at a temperature of 45 °C. The solution was stirred under magnetic stirring (750 rpm) (Ika, Staufen, Germany) for 30 min at the same temperature. Then, the solution was kept for 1.5 h in a water bath at 37 °C. The lipid systems were extruded 15 times by passage through a polycarbonate membrane of 100 nm pore size (Mini-Extruder Extrusion Technique, Avanti Polar Lipids, Inc., Alabaster, AL, USA) for SPC and a 200 nm pore size for SPC-3.

### 4.4. Physico-Chemical Characteristics of Nano-Systems

#### 4.4.1. Mean Particle Size, Zeta Potential, and Polydispersity

These parameters were determined by the dynamic light scattering method using a Zetasizer Nano (Malvern Instrument, Malvern, UK). The size (hydrodynamic diameter, nm) was calculated using the Einstein–Stokes relation *D = k_B_T/3πηx*, where *D* is the diffusion coefficient, *k_B_* is the Boltzmann constant, *T* is the absolute temperature, *η* is the viscosity, and *x* is the average hydrodynamic diameter of the nanoparticles. All obtained samples were diluted 20-fold. The diffusion coefficient was determined in triplicate for each sample.

#### 4.4.2. Transmission Electron Microscopy (TEM)

TEM was used to image the size and to reveal the morphology. TEM images were obtained using a Hitachi HT7700 (Exalens microscope, Tokyo, Japan). The images were acquired at an accelerating voltage of 100 keV. The samples were diluted up to 1000 times and added to a 300-mesh copper grid with continuous carbonformvar support films.

#### 4.4.3. Encapsulation Efficiency (*EE*, %) and Loading Capacity (*LC*, %)

*EE* and *LC* were determined by ultracentrifugation followed by the spectrophotometric assessment of the BChE-IRD concentration. A solution of IRD-BChE-loaded nanoparticles (0.3 mL) was placed in vivaspin 500 Centrifugal Concentrator 1000 kDa (Sartorium Stedim Biotech GmBH, Goettingen, Germany) and centrifuged at 3000 rpm for 5 min and at 7000 rpm for 15 min on a MiniSpin plus centrifuge (Eppendorf AG, Hamburg, Germany) to separate unencapsulated BChE-IRD from the polymersomes and liposomes, respectively. The concentration of compounds was determined by UV absorbance, using a Perkin Elmer Lambda 35 spectrophotometer (Perkin Elmer Instruments, Shelton, CT, USA): for IRD-BChE, this was at 778 nm (ε = 97,869 M^–1^ cm^–1^, 10 mM Tris/HCl buffer, pH 7.4 [15]). *EE*, %, and *LC*, %, were calculated using the following Equations (2) and (3):

(2) $$EE\left(\%\right)=\frac{Total\;amount\;of\;enzyme-Free\;enzyme}{Total\;amount\;of\;enzyme}\times 100\%$$

(3) $$LC\;(\%)=\frac{Total\;amount\;of\;enzyme-Free\;enzyme}{Total\;amount\;of\;copolymer\;or\;lipid}\times 100\%$$

#### 4.4.4. Nano-Compartment Membrane Permeability

This parameter was monitored by dialysis, recording the release of the IRD-BChE enzyme from polymersomes and liposomes. The Spectra/Por^®^Float-A-Lyzer^®^G2 Dialysis Device 1000 kDa (Spectrum Laboratories, Inc., Rancho Dominguez, CA, USA), containing BChE-IRD-loaded nano-systems (1.6 mL), was placed inside dialysis bags (Biotech CE Tubing, 12,000 Da, Sigma-Aldrich) containing 10 mL Tris/HCl buffer (0.01 M), pH 7.4, with stirring. This dialysis bag was placed in thermostatic container with Tris/HCl buffer (0.01 M), pH 7.4, 37 °C with a stirring speed of 150 rpm to control the release of nanoparticle components that may interfere with the detection of IRD-BChE release. At specified intervals, 0.6 mL samples were taken from the dialysis bag medium and were centrifuged, first, in Vivaspin 500 Centrifugal Concentrator 1000 kDa (Sartorium Stedim Biotech GmBH, Goettingen, Germany) and then in a Nanosep centrifugal device 3 K Omega (Pall Corporation, New York, NY, USA) at 7000 rpm 15 min at MiniSpin plus centrifuge (Eppendorf AG, Hamburg, Germany) to fully separate the nanoparticles components from the released IRD-BChE. The absorbance was measured using a Perkin Elmer Lambda 35 spectrophotometer (Perkin Elmer Instruments, Shelton, CT, USA). The samples were analyzed in triplicate.

### 4.5. Kinetic Studies of Free BChE and BChE-Containing Nano-Systems

The IRD-BChE activity with BTC as substrate was determined at 25 °C, in 10 mM Tris/HCl buffer, pH = 7.4, by the Ellman method [52]. Steady-state kinetics were recorded at a 412 nm for 120 s using the thermostated double-beam spectrophotometer TUV9DCS (SILab). For all nano-systems, the IRD-BChE–tetramer concentration in the cuvette was 0.2 nM and the concentration in active sites was 0.8 nM. The calculations of the catalytic parameters (*K_m_*, *V_max_*, *K_ss_*, and *b*) were performed using the Radic equation (Equation (4)) [30,52,53] in Origin 8.5 software (OriginLab Co., Northampton, MA, USA).

(4) $$v=\left(\frac{k_{\mathrm{cat}}\left[E\right]}{1+K_{m}/\left[S\right]}\right)\left(\frac{1+{b\left[S\right]/K}_{ss}}{1+{\left[S\right]/K}_{ss}}\right)$$

The IRD-BChE activity with phenylacetate (PhA)/aspirin as the substrate was determined at 25 °C in 10 mM Tris/HCl buffer, pH = 7.4, supplemented with 10 mM CaCl_2_ for aspirin according to Masson et al. [54]. CaCl_2_ acts as an activator of the enzyme. The stock solutions of PhA/aspirin (purity ≥ 90%, Sigma-Aldrich, Oakville, ON, Canada) were prepared in methanol. The final methanol concentration in the cuvette was 5% (*v*/*v*). Steady-state kinetics were recorded at a 270 nm for PhA and 300 nm for aspirin during 120 s, using thermostated double-beam spectrophotometer TUV9DCS (SILab, Shanghai, China). For all nano-systems, the IRD-BChE–tetramer concentration in the cuvette was 1 nM for PhA and 1.6 nM for aspirin, and the concentration in active sites was 4 nM for PhA assays and 6.25 nM for aspirin assays. For these substrates, the calculations of catalytic parameters (*K_m_*, *V_max_*) were performed using the Michaelis–Menten equation (Equation (1)) in Origin software (OriginLab Co., Northampton, MA, USA). According to the kinetic principles of enzymatic reactions in nano-compartments containing a significantly higher concentration of enzyme than substrate and described in our work [55], the catalytic parameters (*k′_cat_* and *K′_m_*) differ from those determined in solution (*k_cat_* and *K_m_*) due to the characteristics of the medium and the limitations of its volume (nano-volume, crowding effect, viscosity of the medium). *K′_m_* is related to *K_m_* in solution as follows: *K_m_* = *K′_m_* + [*E*]. The rate of the enzymatic reaction at a low concentration of the substrate and a high concentration of the enzyme itself is expressed by the following equation:

(5) $$v=\frac{{k^{\prime }}_{\mathrm{cat}}}{{(K^{\prime }}_{m}+\left[E\right])}\left[E\right]\left[S\right]$$

## Figures and Tables

**Table 1:** Dynamic light scattering data for IRD-BChE-loaded nano-systems (nano-scavengers) in 10 mM Tris buffer, pH = 7.4, 25 °C; Z_aver_ is the mean size, size is hydrodynamic diameter, PDI is polydispersity index, ξ or zeta potential is electrokinetic potential, EE is encapsulation efficiency, LC is loading capacity, liposomes-1 is based on phosphatidylcholine, liposomes-2 is based on hydrogenated phosphatidylcholine, and C_BChE-IRD_ = 0.47 µM (0.16 mg/mL). Data are mean values ± SE of triplicate measurements.

Nanoscavengers	Z_aver_, nm	Size, nm		PDI	ξ, mV	EE, %	LC, %
		Int.	Num				
BChE-polymersomes-1	126 ± 1	122 ± 16	68 ± 15	0.17 ± 0.02	−7.8 ± 0.5	88 ± 1	2.8 ± 0.1
BChE-polymersomes-1 *	106 ± 1	91 ± 7	44 ± 9	0.33 ± 0.01	−8.1 ± 0.4	-	-
BChE-polymersomes-2	127 ± 1	122 ± 19	79 ± 19	0.15 ± 0.01	−32.8 ± 0.3	95 ± 1	3.0 ± 0.1
BChE-polymersomes-2 *	124 ± 1	122 ± 18	68 ± 15	0.14 ± 0.02	−29.5 ± 0.3	-	-
BChE-polymersomes-3	126 ± 2	144 ± 6	70 ± 4	0.22 ± 0.01	−10.6 ± 1.4	92 ± 1	3.0 ± 0.4
BChE-polymersomes-3 *	124 ± 2	142 ± 14	38 ± 8	0.32 ± 0.02	−9.0 ± 1.0	-	-
BChE-polymersomes-4	100 ± 2	91 ± 12	51 ± 12	0.21 ± 0.01	−7.3 ± 0.6	95 ± 2	3.0 ± 0.1
BChE-polymersomes-4 *	125 ± 2	122 ± 12	59 ± 12	0.25 ± 0.01	−11.8 ± 1.8	-	-
BChE-liposomes-1	130 ± 1	145 ± 1	91 ± 3	0.09 ± 0.01	−19.9 ± 0.4	85 ± 3	1.1 ± 0.04
BChE-liposomes-1 *	-	91 ± 4; 396 ± 63	59 ± 13	0.54 ± 0.01	−16.1 ± 0.8	-	-
BChE-liposomes-2	145 ± 1	161 ± 1	107 ± 3	0.11 ± 0.01	−17.8 ± 0.2	77 ± 3	1.0 ± 0.04
BChE-liposomes-2 *	Not stable						

* Stability for 2 months at 4 °C.

## Data Availability

The original contributions presented in this study are included in the article/Supplementary Materials. Further inquiries can be directed to the corresponding authors.

## Supplementary Materials

The following supporting information can be downloaded at: https://www.mdpi.com/article/10.3390/ijms27010190/s1.

## Abbreviations

The following abbreviations are used in this manuscript:

BChE	Butyrylcholinesterase
BTC	Butyrylthiocholine iodide
E	Enzyme
EE	Encapsulation efficiency
IRD	Infra-red dye
LC	Loading capacity
OP	Organophosphate
PC	Phosphatidylcholine
PEG-*b*-PPS	Polyethyleneglycol–polysulfide
PhA	Phenyl acetate
S	Substrate
