# Supplementary material for: Butyrylcholinesterase-Loaded Liposomes and Polymersomes: Catalytic Parameters for Three Types of Substrates
Source: Int J Mol Sci. 2025 Dec 24;27(1):190. doi: 10.3390/ijms27010190 (PMC12785985; doi:10.3390/ijms27010190)
Supplement: Supplementary file 1 [file ijms-27-00190-s001.zip › ijms-4018449-supplementary.pdf]

Supplementary Material

for

Butyrylcholinesterase-loaded liposomes and polymersomes: catalytic parameters for three types of substrates

Zukhra Shaihutdinova[1,2], Svetlana Batasheva[1], Patrick Masson[1,*] and Tatiana Pashirova[1,2,*]

[1] Institute of Fundamental Medicine and Biology, Kazan Federal University, 18 Kremlyovskaya St., 420008 Kazan, Russia, pmasson@kpfu.ru

[2] Arbuzov Institute of Organic and Physical Chemistry, FRC Kazan Scientific Center, Russian Academy of Sciences, Arbuzov Str., 8, 420088 Kazan, Russia, pashirova@iopc.ru

* Correspondence: masson.pym@gmail.com and; tatyana_pashirova@mail.ru

Contents

Figure S1. TEM images of BChE-loaded liposomes and polymersomes S3

Figure S2. Screenshots of size distribution using the intensity and number parameters of BChE-loaded liposomes S4

Figure S3. Screenshots of size distribution using the intensity and number parameters of BChE-loaded polymersomes S5

Figure S4. Screenshots ofzeta-potential of BChE-loaded nanoparticles S6

Figure S5. Screenshots of size distribution using the intensity and number parameters of BChE-loaded polymersomes after 2 month storage S7

Figure S6. Screenshots of zeta-potential of BChE-loaded polymersomes after 2-month storage S8

Figure S7. UV Absorbance spectra of BChE after ultracentrifugation of BChE-loaded nanoparticles S9

Figure S8. UV Absorbance spectra of BChE released from nanoparticles S10

Figure S9. Progressive curves of butyrylthiocholine hydrolysis by BChE-loaded nanoparticles: freshly prepared and stored S11

Figure S10. Progressive curves of butyrylthiocholine hydrolysis by BChE-loaded nanoparticles S12

Figure S11. Progressive curves of phenyl acetate hydrolysis by free BChE and BChE-loaded polymersomes-1 S13

Figure S12. Progressive curves of aspirin hydrolysis by free BChE and BChE-loaded polymersomes-1 S14

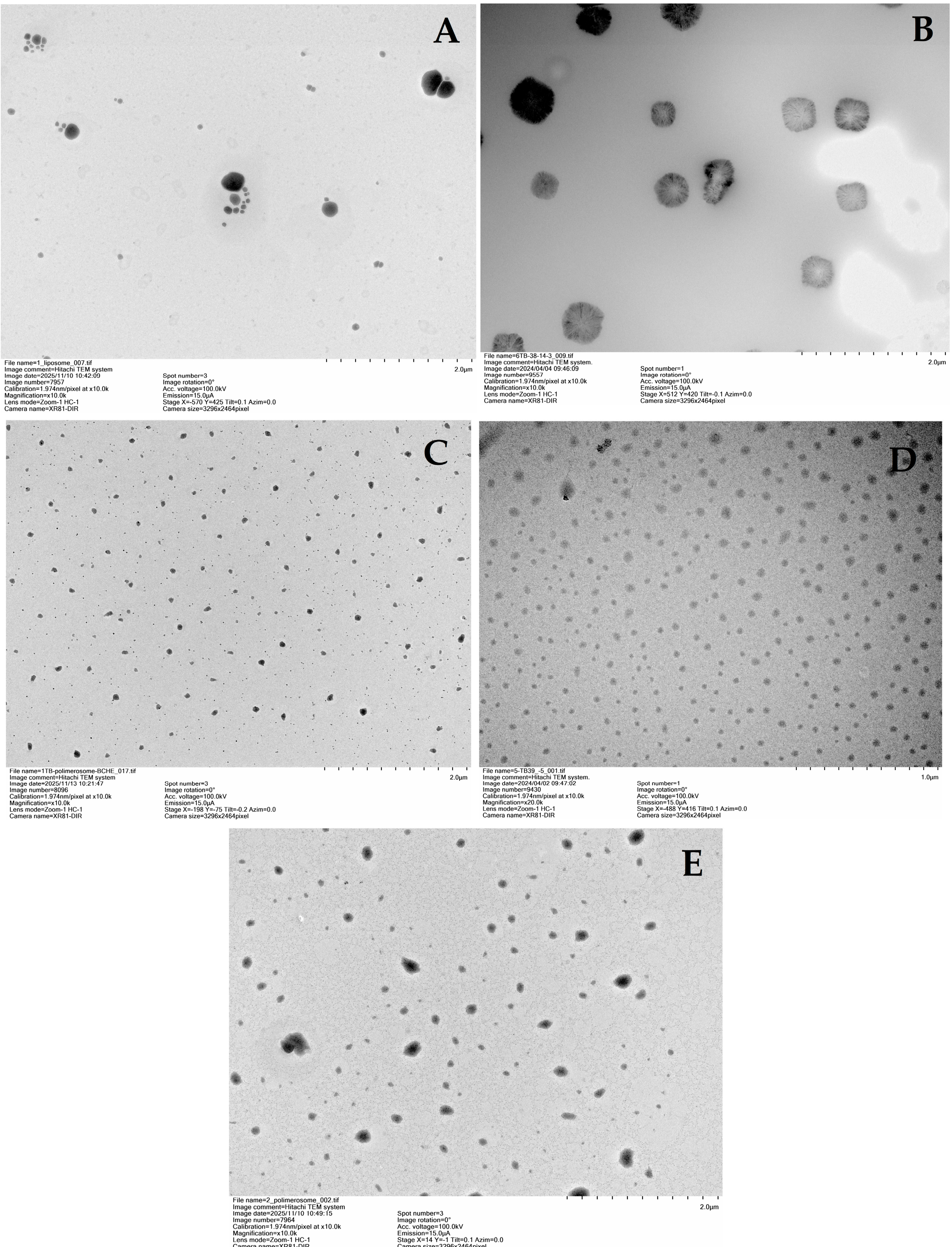

Figure S1. TEM of BChE-containing (A) PEG-liposomes after 2 month storage at 4ºC and (B-E) PEG-*b*-PPS-polymersomes: (B) PEG-*b*-PPS-1; (C) PEG-*b*-PPS-2; (D) PEG-*b*-PPS-3; (E) PEG-*b*-PPS-4; dilution of solution 1000 times, 25 °C.

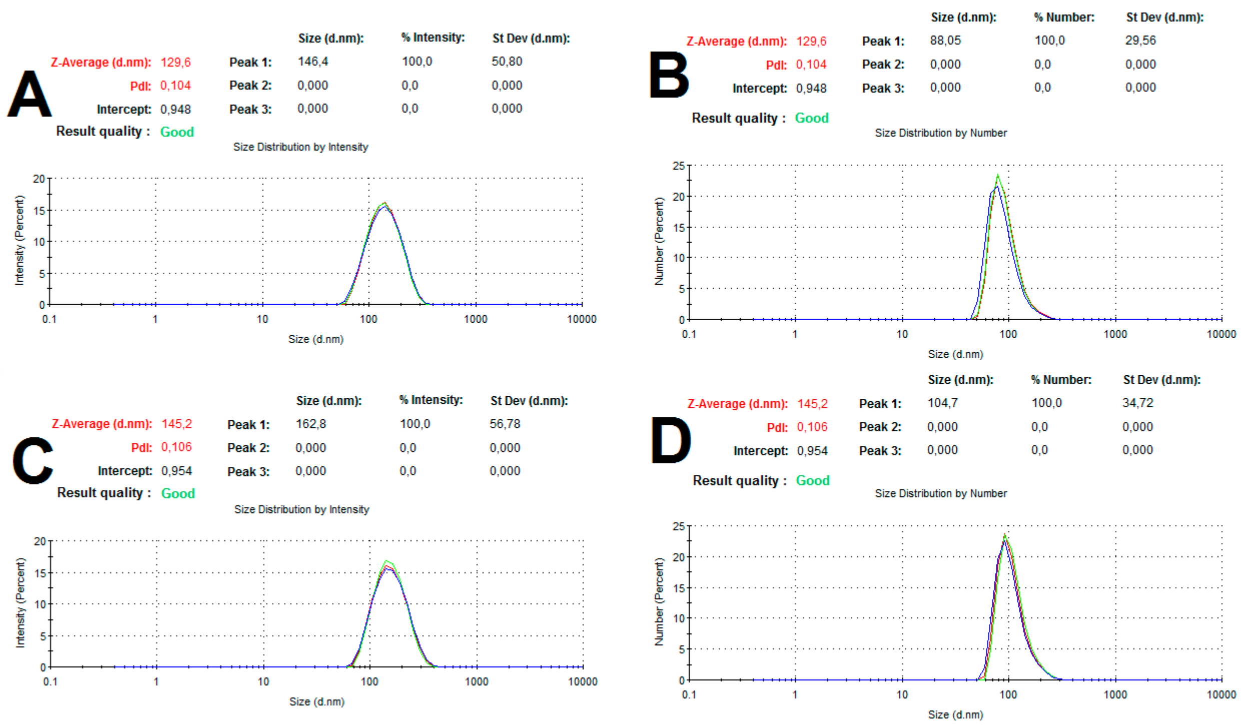

Figure S2. Screenshots of size distribution using the intensity (A,C) and number parameters (B,D) of BChE-loaded (A, B) PEG-liposomes-1 and (C, D) PEG-liposomes-2 in 10 mM Tris/HCl buffer, pH = 7.4, 25 °C.

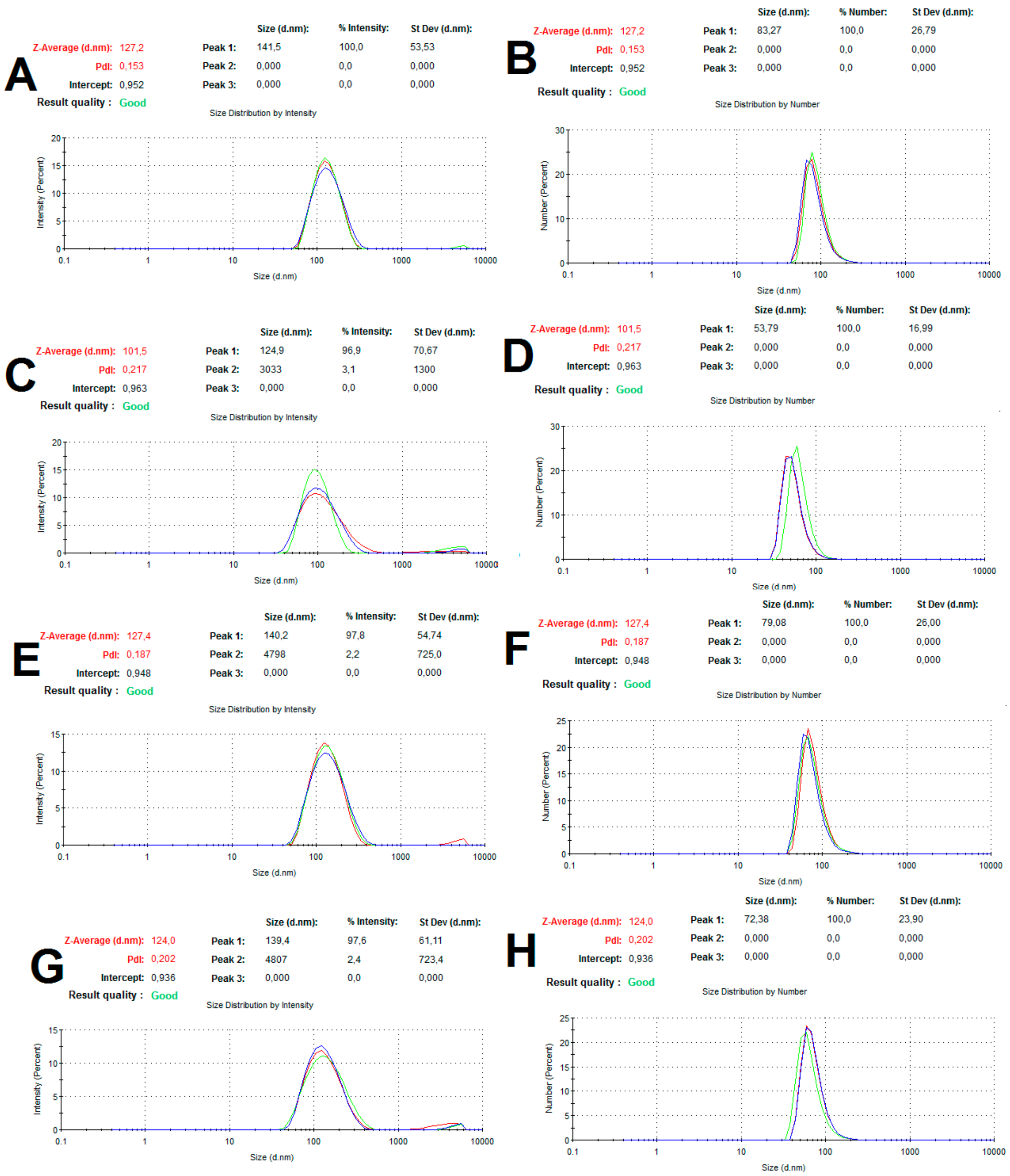

Figure S3. Screenshots of size distribution using the intensity (A,C,E,G) and number parameters (B,D,F,H) of BChE-loaded (A,B) polymersomes-2; (C,D) polymersomes-3; (E,F) polymersomes-1; (G,H) polymersomes-4 in 10 mM Tris/HCl buffer, pH = 7.4, 25 °C.

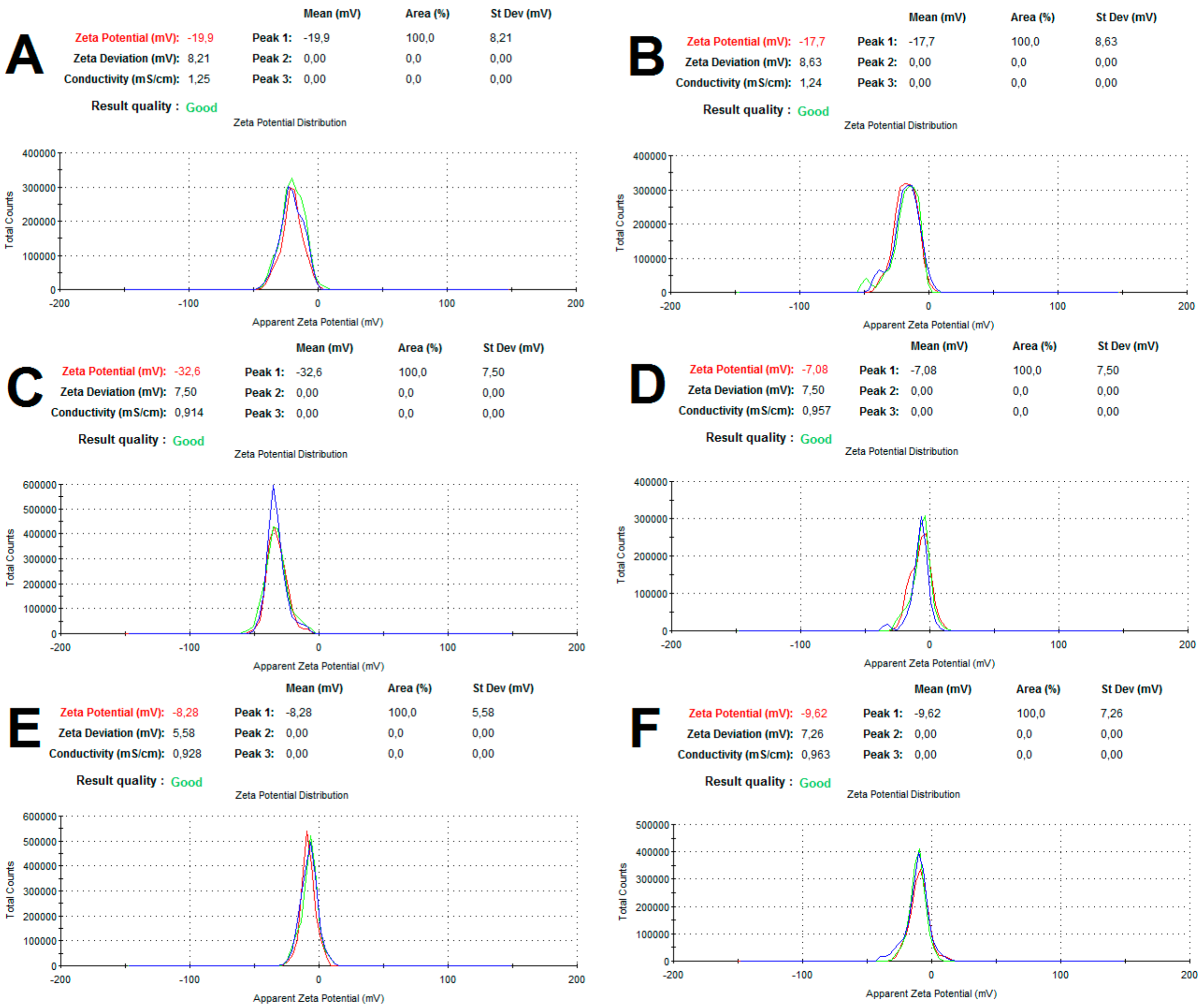

Figure S4. Screenshots of zeta-potential of BChE-loaded (A) PEG-liposomes-1; PEG-liposomes-2 (B), (C) polymersomes-2; (D) polymersomes-3; (E) polymersomes-1; (F) polymersomes-4; in 10 mM Tris/HCl buffer, pH = 7.4, 25 °C.

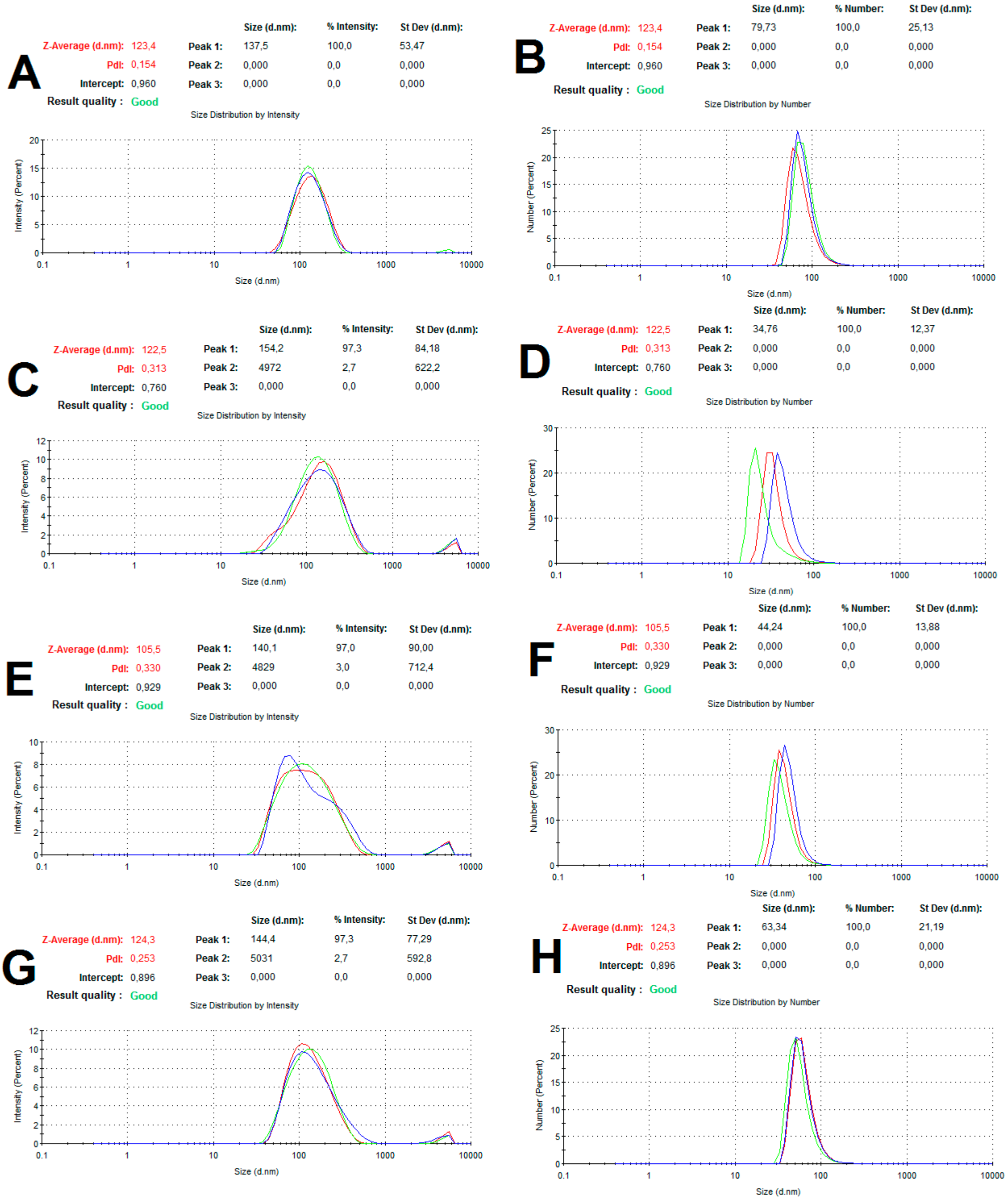

Figure S5. Screenshots of size distribution using the intensity (A,C,E,G) and number parameters (B,D,F,H) of BChE-loaded (A,B) polymersomes-2; (C,D) polymersomes-3; (E,F) polymersomes-1; and (G,H) polymersomes-4 after 2-month storage at 4ºC in 10mM Tris/HCl buffer, pH = 7.4, 25 °C.

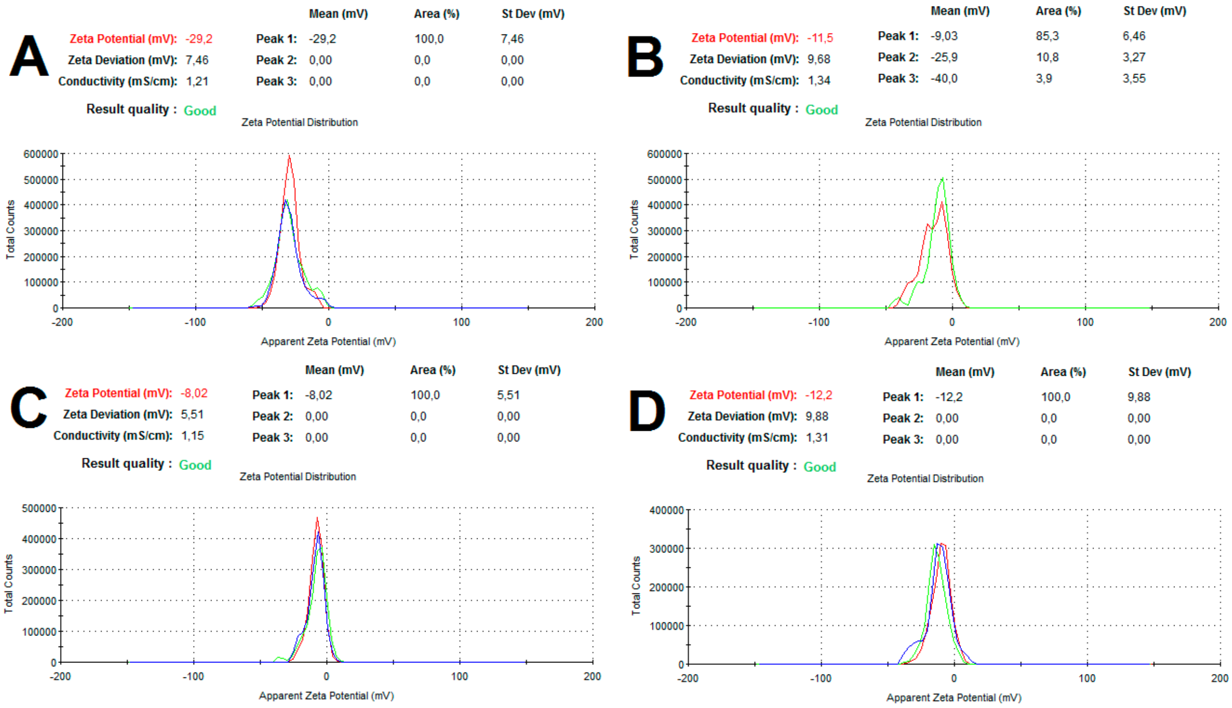

Figure S6. Screenshots zeta-potential of BChE-loaded (A) polymersomes-2; (B) polymersomes-3; (C) polymersomes-1; and (D) polymersomes-4 after 2 month storage at 4ºC in 10 mM Tris/HCl buffer, pH = 7.4, 25 °C.

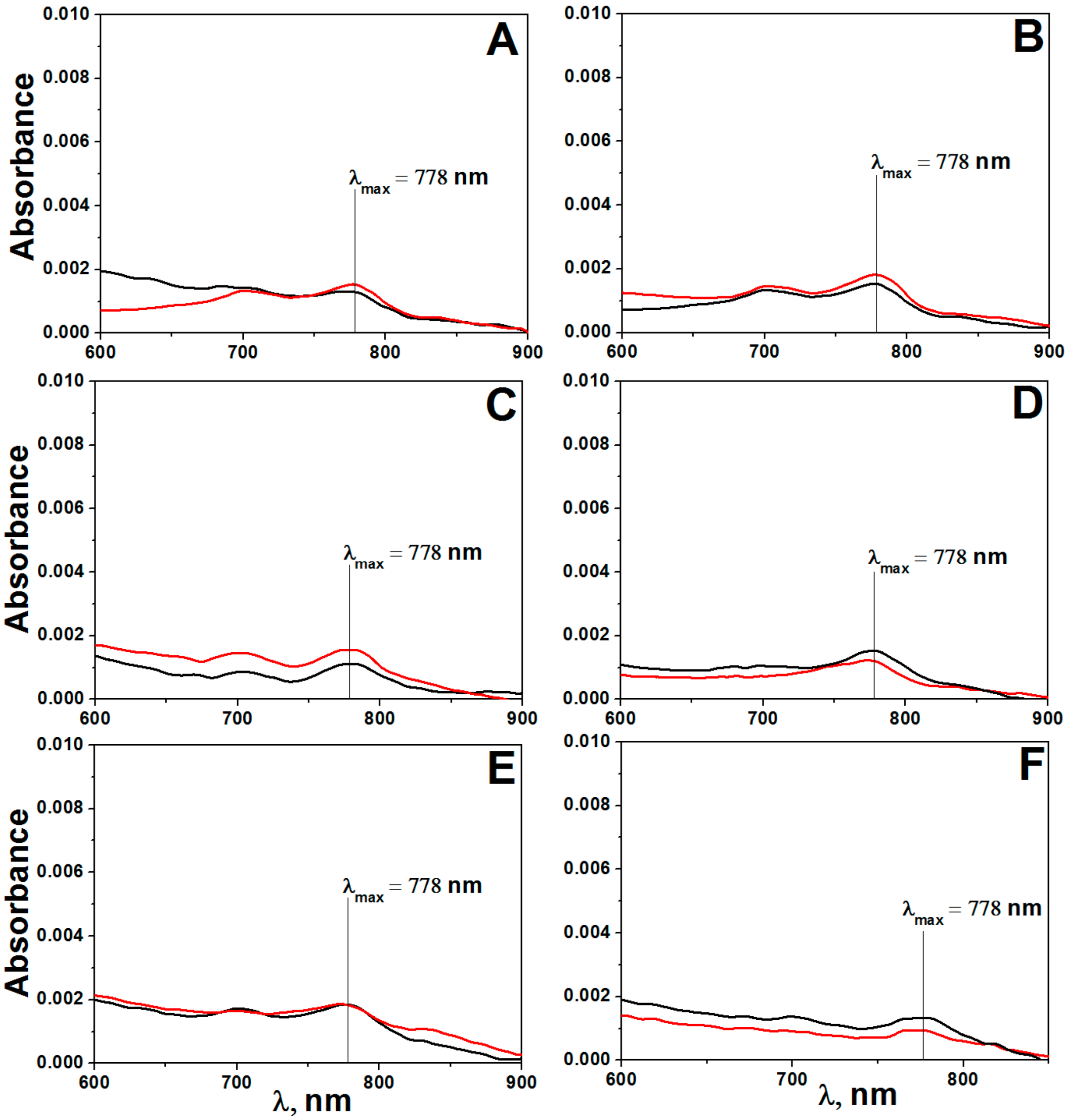

Figure S7. UV Absorbance spectra of BChE after ultracentrifugation of BChE-loaded (A) PEG-liposomes-1; (B) PEG-liposomes-2, (C) polymersomes-2; (D) polymersomes-3; (E) polymersomes-1; (F) polymersomes-4 in 10mM TRIS/HCl buffer, pH=7.4, 25°C.

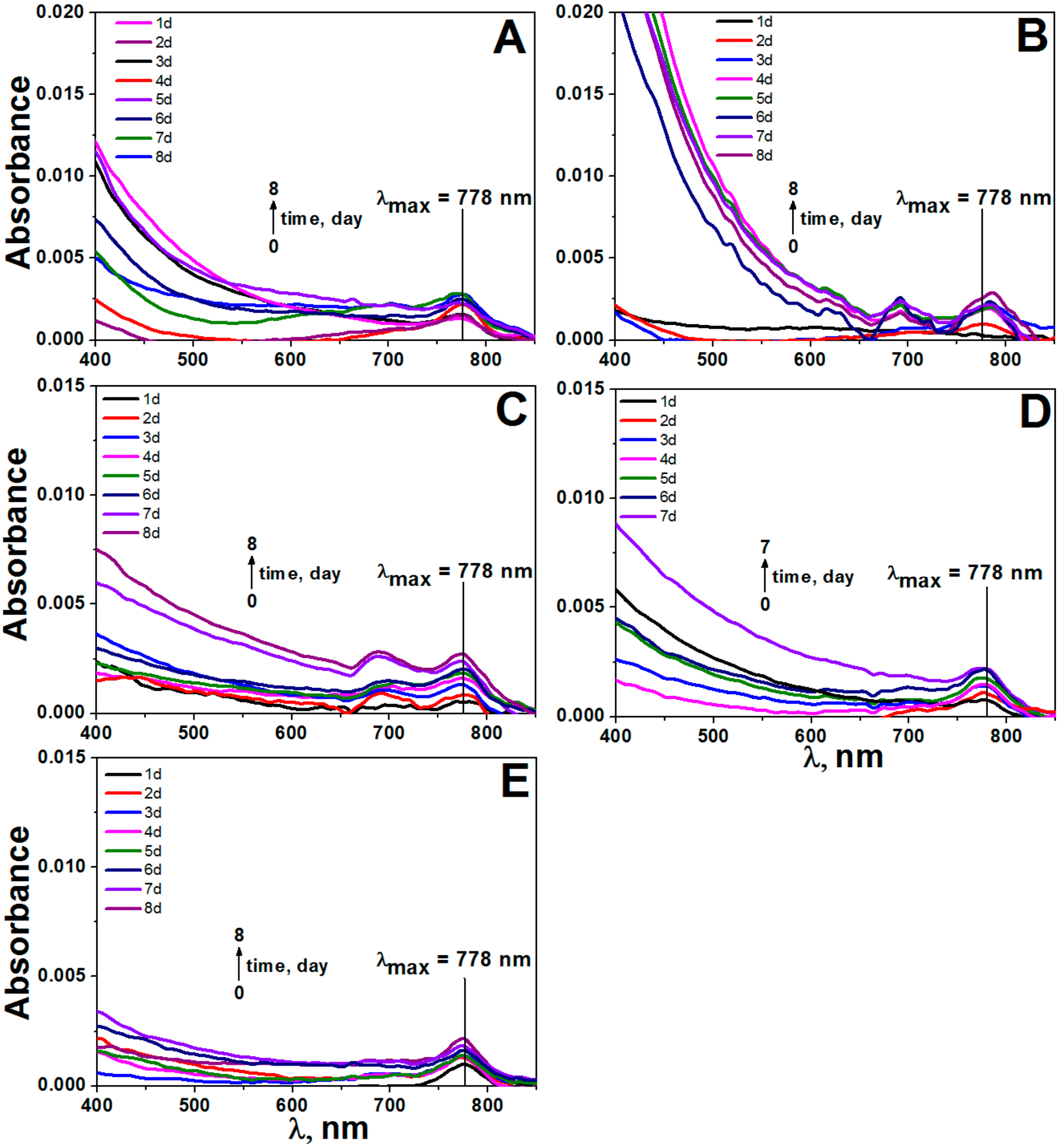

Figure S8. UV Absorbance spectra of BChE released from A) PEG-liposomes-1; (B) PEG-liposomes-2, (C) polymersomes-2; (D) polymersomes-3; (E) polymersomes-1; (F) polymersomes-4, monitored by dialysis method during time, 10 mM Tris/HCl buffer, pH=7.4, 25°C.

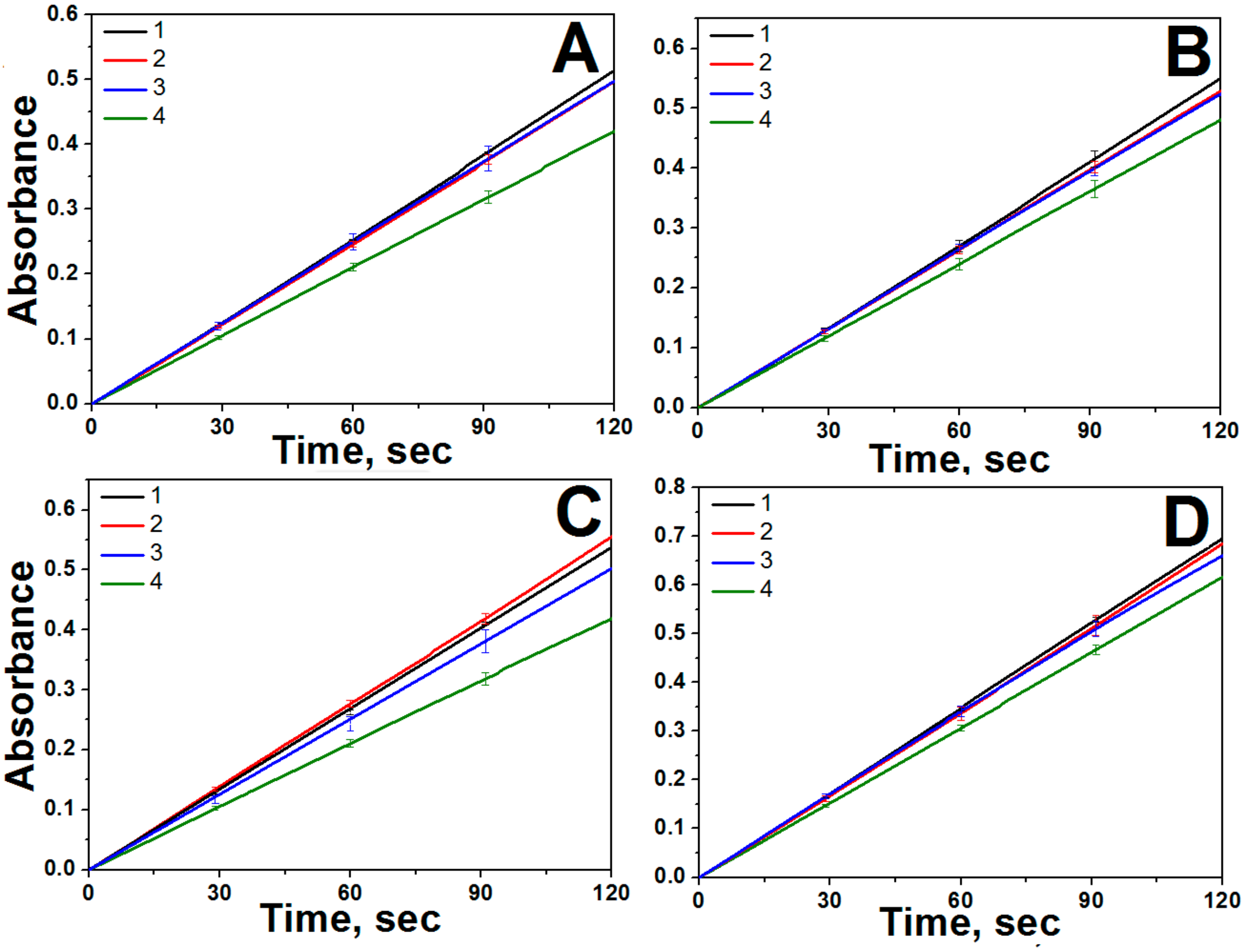

Figure S9. Progress curves of butyrylthiocholine hydrolysis by BChE-loaded polymersomes-2 (A), polymersomes-4 (B), polymersomes-1 (C), polymersomes-3 (D) of freshly prepared (1), stored 2 months at +4 ºC (2), stored 1 day at room temperature (3), and stored 3 days at room temperature (4), C_{BChE} = 0.2 nM, $C_{active\ sites\ of\ BChE}$= 0.8 nM, Tris/HCl buffer (10 mM), pH = 7.4, 25 °C. Data are mean values ± SE of triplicate measurements.

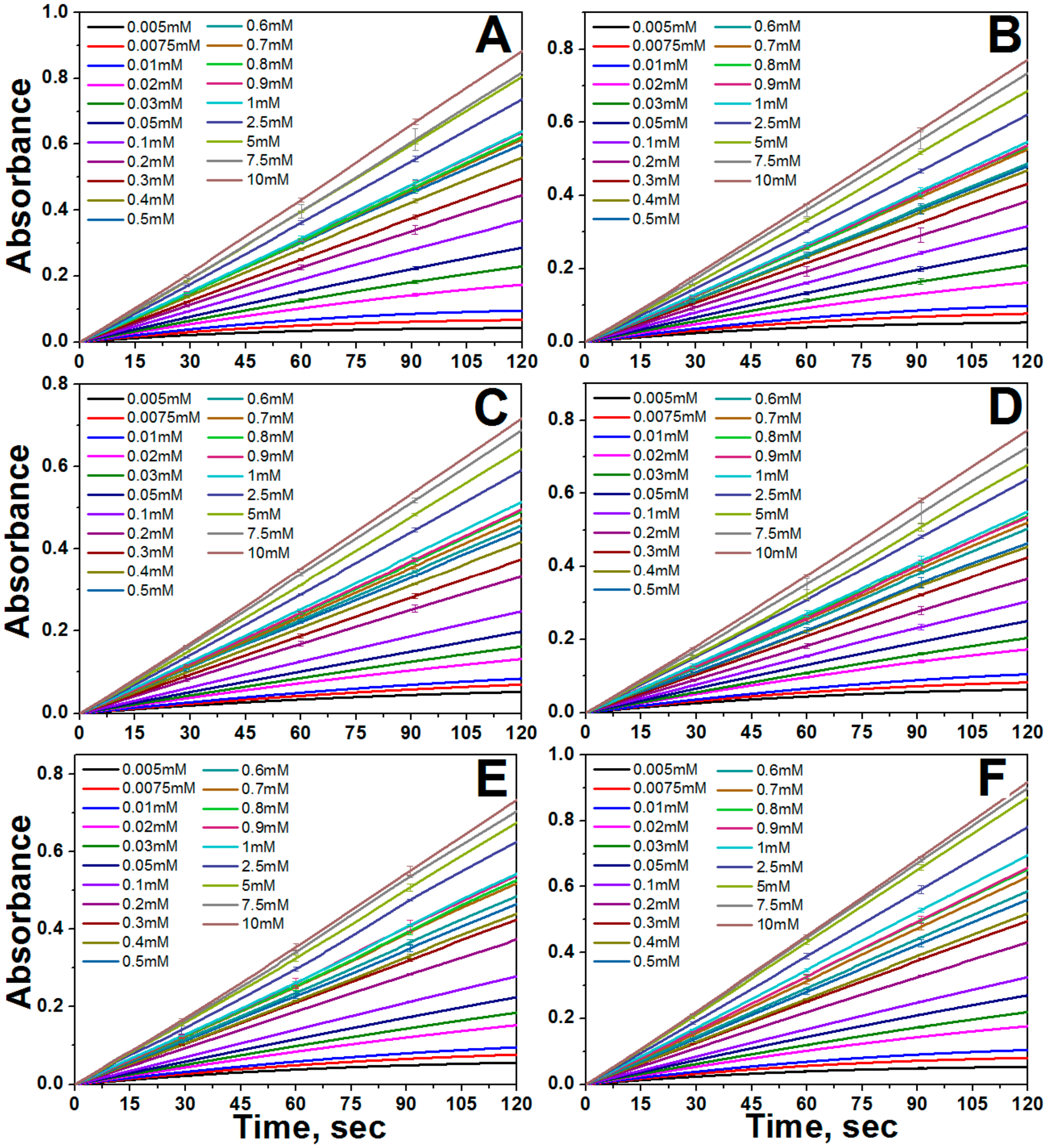

Figure S10. Progress curves of butyrylthiocholine hydrolysis by BChE-loaded A) PEG-liposomes-1; (B) PEG-liposomes-2, (C) polymersomes-2; (D) polymersomes-3; (E) polymersomes-1; (F) polymersomes-4, C_{BChE} = 0.2 nM, $C_{active\ sites\ of\ BChE}$= 0.8 nM, Tris/HCl buffer(10 mM), pH = 7.4, 25 °C. Data are mean values ± SE of triplicate measurements.

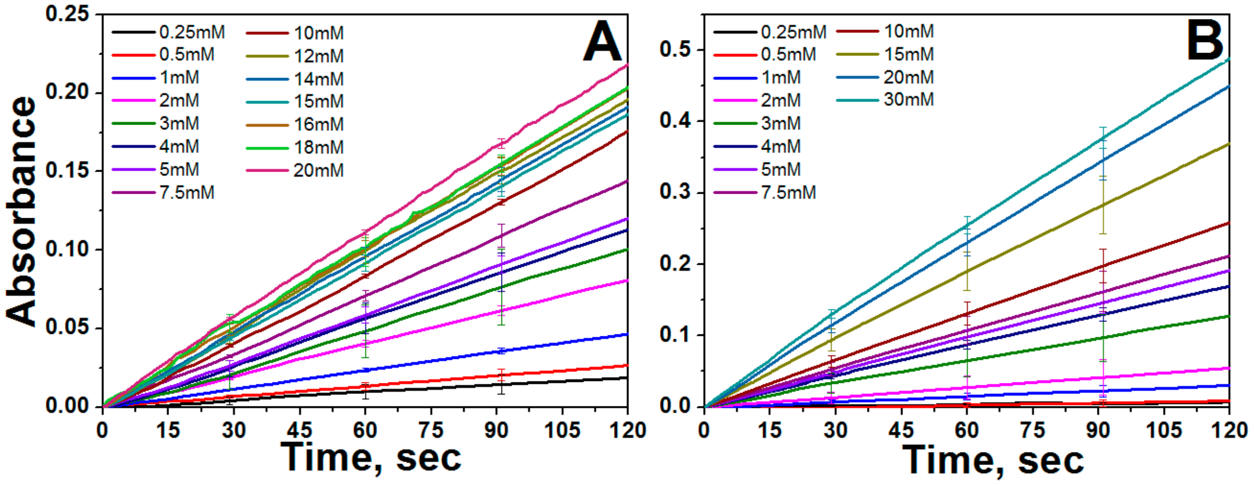

Figure S11. Progress curves of PhA hydrolysis by (A) free BChE and (B) BChE-loaded polymersomes-1, $C_{BChE\ tetramer}$ = 1 nM, $C_{active\ sites\ of\ BChE}$= 4 nM, Tris/HCl buffer (10 mM), pH = 7.4, 5% of methanol, 25 °C. Data are mean values ± SE of triplicate measurements.

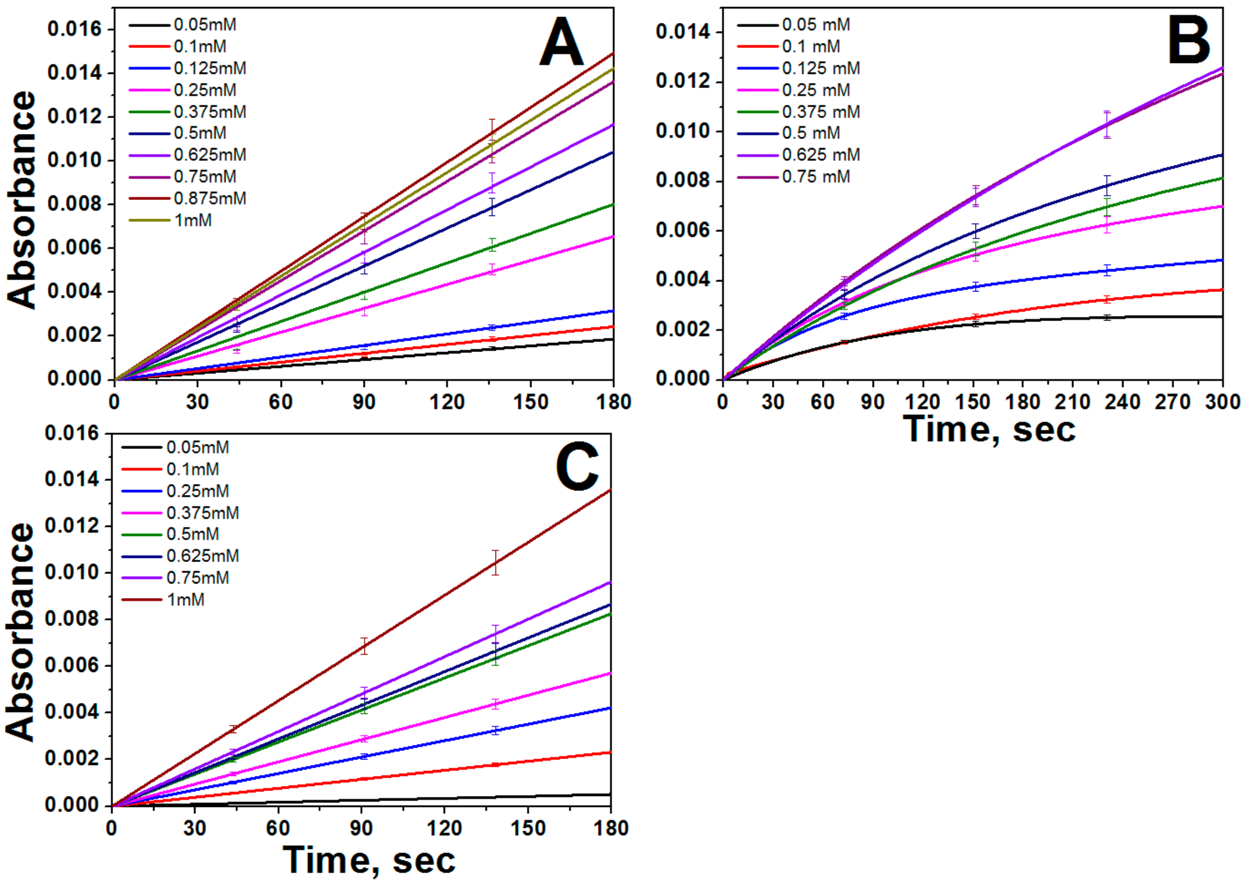

Figure S12. Progress curves of Aspirin hydrolysis by (A) free BChE and (B,C) BChE-loaded polymersomes in 10 mM TRIS/HCl buffer pH 7.4 with (A, B) 10 mM $CaCl_2$ and (C) 100 mM $CaCl_2$ Tris/HCl buffer pH 7.4, 5% of methanol, $C_{BChE\ tetramer}$ = 1.6 nM, $C_{active\ sites\ of\ BChE}$= 6.25 nM, 25 °C. Data are mean values ± SE of triplicate measurements.
